# Direct flow cytometry measurements reveal a fine-tuning of symbiotic cell dynamics according to the host developmental needs in aphid symbiosis

**DOI:** 10.1038/srep19967

**Published:** 2016-01-29

**Authors:** Pierre Simonet, Gabrielle Duport, Karen Gaget, Michèle Weiss-Gayet, Stefano Colella, Gérard Febvay, Hubert Charles, José Viñuelas, Abdelaziz Heddi, Federica Calevro

**Affiliations:** 1 UMR203 BF2I, Biologie Fonctionnelle Insectes et Interactions, INRA, INSA de Lyon, Université de Lyon, F-69621 Villeurbanne, France; 2 UMR5534, Centre de Génétique et de Physiologie Moléculaire et Cellulaire, Université Lyon 1, CNRS, F-69622 Villeurbanne, France

## Abstract

Endosymbiotic associations constitute a driving force in the ecological and evolutionary diversification of metazoan organisms. Little is known about whether and how symbiotic cells are coordinated according to host physiology. Here, we use the nutritional symbiosis between the insect pest, *Acyrthosiphon pisum*, and its obligate symbiont, *Buchnera aphidicola*, as a model system. We have developed a novel approach for unculturable bacteria, based on flow cytometry, and used this method to estimate the absolute numbers of symbionts at key stages of aphid life. The endosymbiont population increases exponentially throughout nymphal development, showing a growing rate which has never been characterized by indirect molecular techniques. Using histology and imaging techniques, we have shown that the endosymbiont-bearing cells (bacteriocytes) increase significantly in number and size during the nymphal development, and clustering in the insect abdomen. Once adulthood is reached and the laying period has begun, the dynamics of symbiont and host cells is reversed: the number of endosymbionts decreases progressively and the bacteriocyte structure degenerates during insect aging. In summary, these results show a coordination of the cellular dynamics between bacteriocytes and primary symbionts and reveal a fine-tuning of aphid symbiotic cells to the nutritional demand imposed by the host physiology throughout development.

Intracellular symbioses (endosymbioses) between prokaryotic and metazoan organisms play a central role in multicellular life, significantly impacting the evolution and shaping the ecology of countless species^1^. In insects, which account for a great proportion of planet biodiversity, the exploitation of the metabolic capabilities of intracellular symbiotic bacteria (endosymbionts) enables the hosts to thrive on nutritionally unbalanced diets such as plant sap, grains, wood or vertebrate blood^2^,^3^,^4^. The sustainability of these endosymbiotic relationships largely relies on the compartmentalization of bacterial endosymbionts into specialized host cells (or organs), called bacteriocytes (or bacteriomes), whose functions are adapted to the tolerance and regulation of symbiotic populations^5^,^6^. A detailed description of the interplay between bacteriocytes and endosymbionts across the host life cycle, and in response to an ever-changing environment, is expected to provide a better understanding of how microorganisms interact with eukaryotic cells, and, in turn, to contribute to the development of novel strategies for controlling pest and disease-vector insects.

The relationship between aphids (Hemiptera: Aphididae) and the gamma-3-proteobacterium *Buchnera aphidicola*, represents the best-studied model among endosymbiotic associations. In the *A. pisum*/*B. aphidicola* interaction, the availability of full genome sequences for both partners allowed considerable advances to be made in the understanding of the molecular basis of symbiotic interactions^7^,^8^,^9^. The two partners are complementary in their genomes for several metabolic pathways, leading to the merger of each organism’s capabilities into a single integrated metabolism^10^. On the one hand, *B. aphidicola* supplies the host with the vitamins and the essential amino acids that the insect cannot synthesize or derive in sufficient amounts from its exclusive diet of the plant phloem sap. On the other hand, aphids provide the symbiont with the non-essential amino acids and core metabolites that the bacterium can no longer produce due to the massive gene losses affecting its central metabolic pathways^4^,^11^. Hence, the mutualism between aphids and *B. aphidicola* has reached such an extent that both partners have become completely interdependent and neither can reproduce in the absence of the other.

Aphids exhibit the particularity of reproducing asexually by viviparous parthenogenesis for much of their life cycle, leading to the development of embryos within maternal ovarioles. This mode of reproduction has a considerable impact on the symbiosis topology in aphids, for instance with the existence of two distinct symbiotic compartments: the maternal bacteriocytes and those contained in the developing embryos. To ensure the sustainability of the symbiotic association, a limited number of symbionts are vertically transmitted from a single maternal bacteriocyte into an adjacent blastula-stage embryo, by a selective exo-/endocytotic process^12^,^13^. The symbionts, initially colonizing the posterior syncytial cytoplasm of the embryo, are then segregated in distinct bacteriocyte cells as embryonic bacteriocyte cellularization proceeds. In late parthenogenetic embryogenesis, these bacteriocytes harboring *B. aphidicola* increase in number and cluster into two regular structures located along the digestive tract of the late embryo^13^,^14^,^15^.

Although considerable progress has been made in describing the maternal transmission of symbiotic bacteria in aphids, the relative dynamics between endosymbionts and bacteriocytes after the embryo/oocyte infection, and over the entire insect life cycle, remain largely unexplored. Over recent decades, the approach most often used to examine symbiont density in insects has been based on the estimation of symbiont gene copy number by quantitative PCR techniques^16^,^17^,^18^,^19^,^20^,^21^,^22^,^23^. However, PCR-based approaches only provide indications of the bacterial load in a given tissue or organism, and there is a need to develop cellular techniques to generate direct information and absolute data on endosymbiont population size and dynamics in symbiotic models. In aphids, a few studies have analyzed the endosymbiont number and the bacteriocyte cell dynamics using microscopy techniques^24^,^25^,^26^. Nevertheless, these histological data are fragmented, not providing an overview of the complete aphid life cycle. These data may also be contradictory when analyzing studies published by different authors.

Over the last decades, flow cytometry has become a valuable tool within the field of environmental microbiology^27^,^28^. This cellular technique has been used to quantify bacterial numbers throughout the entire range of growth phases under a large variety of physiological conditions and for microbes living in a wide diversity of habitats: soil^29^, water^30^ or food^31^.

The flow cytometry ability to generate a single stream of cells by hydrodynamic focusing offers the advantage to simultaneously analyze, in a high-throughput way, multiple parameters at a single-cell level. The extreme sensitivity, the high degree of reproducibility, the rapidity and the high numerical resolution of flow cytometry considerably improved the accuracy of the detection and counting of micro-organisms in complex samples, irrespective of their culturability, compared to the traditional used methods (i.g. manual counting by microscopy, cultivation-dependent or indirect molecular methods). Nevertheless, in spite of its wide use in environmental applications, the flow cytometry technique has never been used to quantify the absolute number of unculturable symbiotic bacteria in insect models.

In the present work we have analyzed, for the first time, the dynamics of symbiotic cells, both at the bacteriocyte and at the primary endosymbiont population levels, during the pea aphid life cycle from the late embryonic to the senescent adult stages. Through the development of a cellular approach based on flow cytometry, new for symbiosis, we were able to quantify the absolute number of *B. aphidicola* cells in the two symbiotic compartments of parthenogenetic aphids: the maternal bacteriocytes and those contained in the embryonic chains, thus taking into account the tissue complexity of this symbiotic model. In parallel, the bacteriocyte tissue dynamics was analyzed in terms of size, number and structure, with a combination of cell imaging and histology techniques. Taken together, these data draw a step-by-step picture of the coordination dynamics between the host cells and the bacteria they house, as dictated by the changing nutritional requirements during the development of a parthenogenetic insect.

## Results

### Identification of *B. aphidicola* endosymbionts using flow cytometry

**Figure 1:**
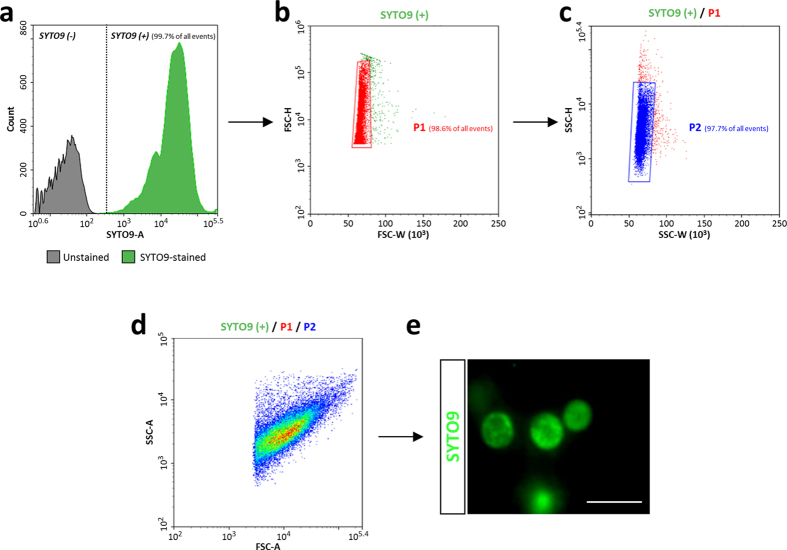
Detection of endosymbionts by flow cytometry analysis. (**a**–**d**) Gating strategy used to analyze *B. aphidicola* cells. (**a**) Fluorescence histogram overlay of unstained and SYTO9-stained samples, with the dotted line referring to the autofluorescence limit. SYTO9 positive events were selected, and debris excluded from the analysis. (**b**) SYTO9 positive events were viewed in a FSC-W/FSC-H plot to select cells homogeneous in size (gate P1) and depict cell doublets/aggregates. (**c**) Events in gate P1 were viewed in a SSC-W/SSC-H plot to select cells homogeneous in granularity (gate P2). (**d**) Cells identified as *B. aphidicola* were represented on density plot (FSC-A/SSC-A), the color gradient indicating increasing density of dots. (**e**) Microscopy image of SYTO9-stained symbionts. Scale bars = 5 μm.

Despite the extensive use of flow cytometry for cell counting in biological applications, no previous studies have used this technique to quantify endosymbionts in symbiotic insects. We have adapted flow cytometry to the aphid model, using an aphid clone housing the *B. aphidicola* endosymbiont and deprived of all other secondary symbionts described in aphid populations (see the Methods section). To develop this novel approach, we took into account the specificity of aphid symbiosis, in which symbiotic bacteria are localized in specific insect cells, the bacteriocytes, which need to be isolated from other aphid tissues. Consequently, to adapt flow cytometry protocols for aphids, we developed a two-step procedure: (i) bacteriocytes were isolated by dissection from fresh aphid tissues and (ii) symbionts were extracted from isolated bacteriocytes by filtration, thereby excluding the presence of eukaryotic cells (on average ten times bigger than *B. aphidicola* cells). Endosymbiont suspensions were then analyzed by flow cytometry, using an adapted sequential gating procedure. To discriminate endosymbiont cells from electronic background noise and debris, we selected the events positively labeled with the SYTO9 green fluorescent nucleic acid stain (Fig. 1a), after confirming that the buffer used to resuspend cells did not generate any misleading signals. The SYTO9 dye, frequently used for bacterial viability analyses, was shown to be well suited for flow cytometry applications in prokaryotic organisms^32^,^33^,^34^,^35^,^36^. It has the advantage of permeating viable cells without requiring repeated washing steps, which result in considerable loss of cells and often interfere with data repeatability and reliability. The SYTO9-positive population, representing 99.7% of total events (Fig. 1a), was then sequentially analyzed following size (FSC-W/FSC-H; Fig. 1b) and granularity (SSC-W/SSC-H; Fig. 1c) parameters. The combination of the FSC-W/FSC-H and SSC-W/SSC-H analyses was performed to exclude cell doublets/aggregates and cells with sizes and granularities fairly divergent from the majority of the cell population. These two filters allowed rejecting only 1.1% and 0.9% of SYTO9-positive events, respectively. This enabled us to gate cells homogeneous in size (P1, Fig. 1b) and granularity (P2, Fig. 1c), most likely belonging to the same cell type. The resulting FSC-A/SSC-A density plot (Fig. 1d) highlighted a unique major cell population only, representing 97.7% of all events initially detected. This result demonstrates the efficiency of the extraction procedure in terms of sample purity, the great homogeneity of the symbiont suspension, and the efficient estimation of *B. aphidicola* population. It is noteworthy that almost all the SYTO9-positive cells had a characteristic globular shape with a diameter ranging from 2 to 5 μm (Fig. 1e), as previously described for *B. aphidicola*^37^,^38^.

**Figure 2:**
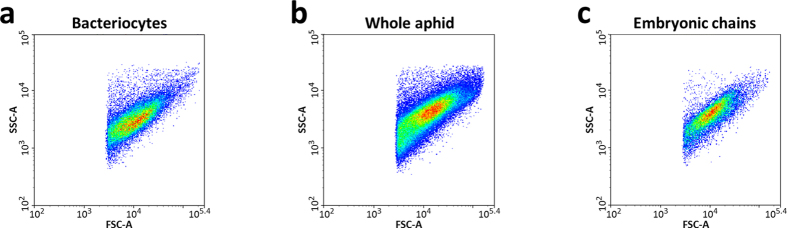
Flow cytometry profiles of endosymbiont populations obtained from different aphid body compartments. Density plots (FSC-A/SSC-A) of *B. aphidicola* cells extracted from isolated bacteriocytes (**a**), whole aphids (**b**), or isolated embryonic chains (**c**), showing the detection of a unique cell population, homogeneous in size and granularity, in all three samples.

Following the validation of the flow cytometry settings on symbionts from isolated bacteriocytes, we applied this methodology to symbiont suspensions extracted by filtration from whole aphids, or isolated from female embryonic chains. We again obtained the characteristic *B. aphidicola* profile described above, with a unique detected cell population in the FSC-A/SSC-A density plots. Moreover, the symbiont populations detected in our samples were very similar in size and granularity (Fig. 2). Likewise, the efficiency of SYTO9 staining and FSC/SSC discrimination were not significantly different between the three samples: isolated bacteriocytes (97.7%, Fig. 2a), whole aphids (96.1%, Fig. 2b) and isolated embryonic chains (96.2%, Fig. 2c). This indicates that the symbiont isolation process does not generate more debris in samples from whole aphids or embryonic chains than from isolated bacteriocytes. Overall, these results demonstrate the efficiency and the reliability of the flow cytometry and gating strategy used here, coupled with our filtration protocols, for identifying and counting *B. aphidicola* cells extracted from fresh aphid tissues.

### Endosymbiont population dynamics during aphid development

**Figure 3:**
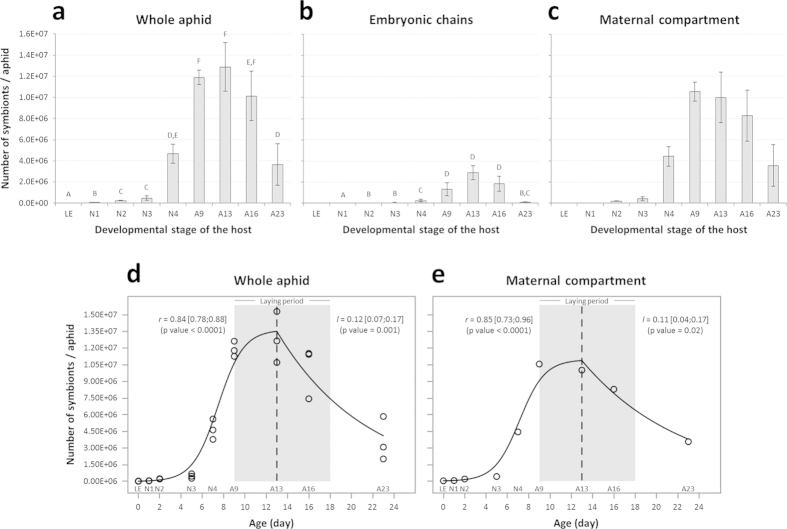
Dynamics of *B. aphidicola* population during aphid development. (**a**,**b**) Variation in the number of symbiont cells, quantified by flow cytometry analysis from whole aphids (**a**), or embryonic chains (**b**), in relation to host developmental stage. Results are reported as means ± SD; n = 3 independent biological replicates per stage (each biological replicate was composed of 10 aphids). Data (after log-transformation) were analyzed by one-way ANOVA followed by a *post hoc* multiple comparisons test (Tukey’s HSD test). Developmental stages labeled with different letters are significantly different (P < 0.05). (**c**) Variation in the number of *B. aphidicola* cells in maternal bacteriocytes, as calculated by subtraction of embryonic chains from whole aphids sample means, in relation to host developmental stage. Results are reported as means ± SD* 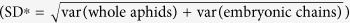. (**d**,**e**) Modeling of *B. aphidicola* population dynamics in whole aphids (**d**) or maternal bacteriocytes (**e**) during aphid development. A logistic model was applied for the first part of the dynamics (n0 fixed), followed by an exponentially decreasing model. The rupture point (vertical dashed line) was visually determined. The predicted value of the logistic model at this rupture point was used to fix Nmax in the exponentially decreasing model for ensuring continuity and improving the estimation of the population decreasing rate. Population growth, *r*, and decreasing rates, *l*, are shown on the figure, with their 95% confidence interval. Abbreviations: LE, late embryos; N1 to N4, nymphal stages from 1 to 4; A9-A23, adult time points from day 9 to day 23. Shaded grey area indicates the laying period.

Using this newly developed flow cytometry protocol, we have estimated the symbiotic bacteria numbers during aphid development. Endosymbiont quantifications were first conducted on whole aphids. This showed that the *B. aphidicola* number varies significantly over the host life cycle (ANOVA test, P < 0.001, Fig. 3a). First, the endosymbiont population drastically increases during nymphal development, multiplying by 460-fold the number of bacteria from the late embryo stage (LE: 2.6 × 10^4^) to the onset of the adult period (A9: 1.2 × 10^7^). Despite the continuous increase, we noticed that the main augmentation occurs at the end of the nymphal phase, between the third nymphal instar (N3: 4.8 × 10^5^) and the A9 time point (A9: 1.2 × 10^7^). After entering the adult phase, the endosymbiont number reaches a stationary phase (A9 to A13) and, finally, we observed a significant drop in endosymbiont numbers during the adult senescent phase (A23: 3.7 × 10^6^). Because the different time points are biologically independent, dynamics modeling allowed only for rough estimations of the exponential growth (*r* = 0.84 ± 0.06 day^−1^) and revealed decreasing rates (*l* = 0.12 ± 0.05 day^−1^) for *B. aphidicola* populations from whole aphids (Fig. 3d).

We next quantified the developmental variation of endosymbionts localized in embryonic chains (Fig. 3b). *B. aphidicola* cell number appeared to also vary over the host life cycle in this symbiotic compartment (ANOVA test, P < 0.001), but with specific dynamics. We observed a first phase of symbiont increase from the first nymphal instar (N1: 1.0 × 10^4^) to the adult stages (A13: 2.9 × 10^6^), corresponding to the period of embryogenesis in aphid ovaries. The endosymbiont number in embryonic chains then significantly decreases during aphid aging (A23: 1.0 × 10^5^), consistent with the observation that old aphid embryonic chains contain fewer embryos than earlier stages (each adult laying 7–8 embryos per day).

As the protocol for flow cytometry analysis of *B. aphidicola* cells was developed starting from isolated bacteriocyte samples, the quantification of endosymbionts localized in maternal bacteriocytes could, in theory, have been directly measured by flow cytometry. However, the process of bacteriocyte isolation and transfer after dissection, prior to the bacteriocyte filtration that is necessary for isolating endosymbiont cells, generates important losses in the bacteriocyte number recovery and, consequently, a great variability in the quantification of *B. aphidicola* cells. Therefore, we have calculated the variation during development of *B. aphidicola* cells in the maternal compartment by subtracting the endosymbiont number of the embryonic compartment from the whole aphid samples (Fig. 3c). As a result, we have characterized a two-phase dynamics in maternal bacteriocytes, with an important increase in endosymbiont number during nymphal development, peaking at the onset of the adult period (LE: 2.6 × 10^4^; A9: 1.1 × 10^7^) and then progressively decreasing until the adult senescent time points (A23: 3.6 × 10^6^). Modeling gave rough estimations (*r* = 0.85 ± 0.11 day^−1^ and *l* = 0.121 ± 0.06 day^−1^) of the rate of exponential growth and then of the decrease of this dynamics (Fig. 3e).

### Dynamics of bacteriocytes during aphid development

The application of flow cytometry to isolated bacteriocytes was not feasible because it requires a minimum of 50,000 cells per sample, which implies taking more than 2,500 synchronized insects (with a mean content of 56 bacteriocytes) per time point. For a three-replicate analysis, a total number of 22,500 aphids would then be required. Moreover, the median size of bacteriocytes is at the higher limits of cell sizes tolerated by the fluidic system of the cytometer used in this study. Instead, the variation of bacteriocyte number during aphid development was measured using microscopy techniques on 20 synchronized aphids for each single time point (see the Methods section).

**Figure 4:**
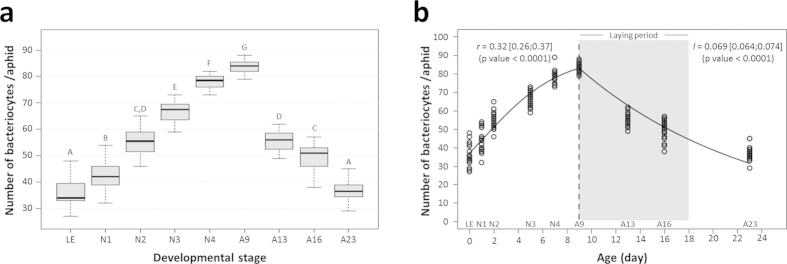
Dynamics of bacteriocyte number during aphid development. (**a**) Variation in the number of bacteriocytes per aphid, in relation to host developmental stage. Results are displayed as box plots where central lines represent the medians, boxes comprise the 25–75 percentiles and whiskers denote the range; n = 20 aphids per stage, for a total number of 180 aphids dissected and analyzed. Data were analyzed by the Kruskal-Wallis test followed by a *post hoc* multiple comparisons test (Wilcoxon rank-sum test with Bonferroni correction). Developmental stages labeled with different letters are significantly different (P < 0.05). (**b**) Modeling of bacteriocyte number dynamics during aphid development. A logistic model was applied for the first part of the dynamics, followed by an exponentially decreasing model. Population growth, *r*, and decreasing rates, *l*, are shown on the figure, with their 95% confidence interval. Shaded grey area indicates the laying period.

**Figure 5:**
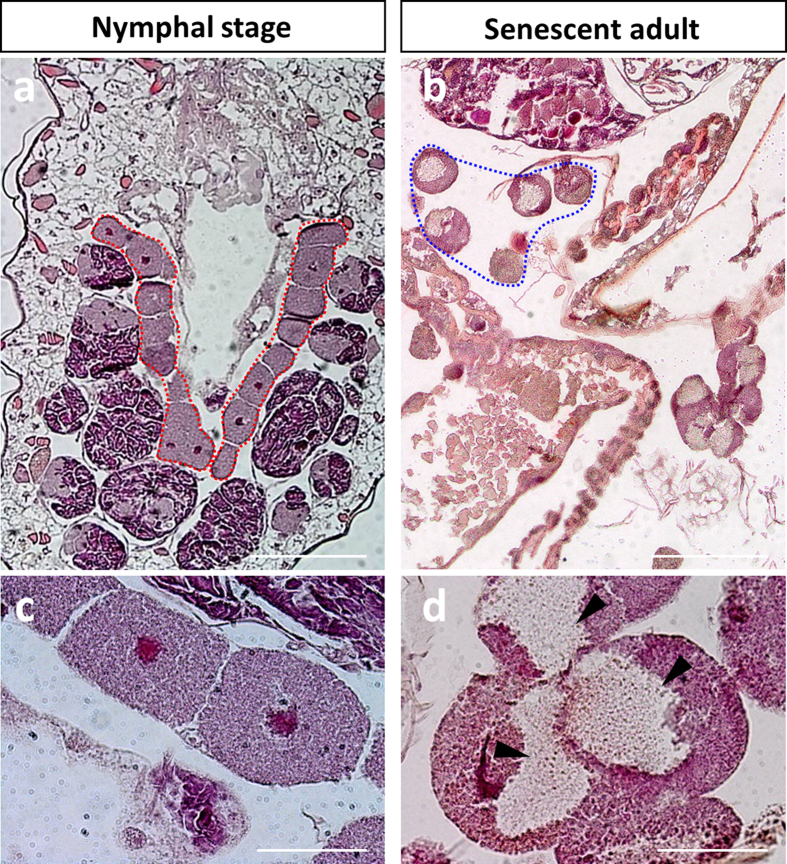
Dynamics of bacteriocyte structures during aphid aging. Comparison of hematoxylin and eosin stainings performed on parthenogenetic N3 nymph (**a**,**c**) and A23 senescent adult (**b**,**d**) sections. The top panels (**a**,**b**) show the general organization of the bacteriocyte structures in the aphid abdomen. Red and blue dotted lines outline the clustering of bacteriocytes into two regular structures surrounded by the embryonic chains, in nymphs, and the degenerating bacteriocyte structures, in senescent aphids, respectively. The bottom panels (**c**,**d**) show representative bacteriocyte cells. Arrowheads show low symbiont-density zones in degenerating bacteriocytes. Scale bars = 200 μm (**a**,**b**) or 50 μm (**c**,**d**).

**Figure 6:**
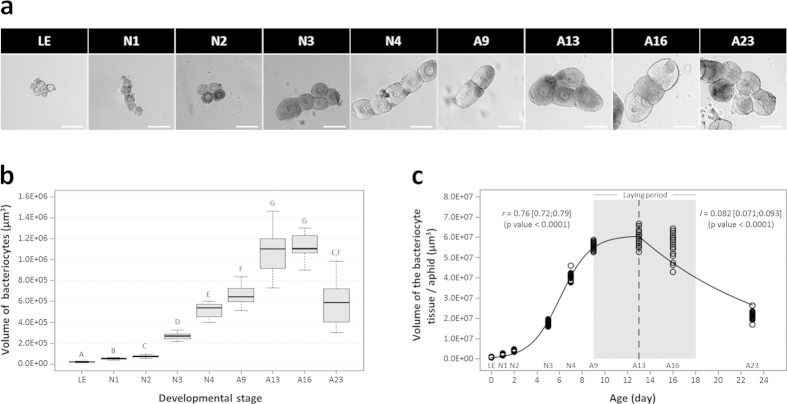
Dynamics of the bacteriocyte size during aphid development. (**a**) Representative microscopy images of bacteriocyte cells at different developmental stages. Scale bars = 100 μm. (**b**) Variation in the volume of bacteriocytes, in relation to aphid developmental stage. Results are displayed as box plots where central lines represent the medians, boxes comprise the 25–75 percentiles and whiskers denote the range; n > 10 aphids per stage, for a total number of more than 4,000 bacteriocytes isolated and analyzed. Data were analyzed by the Kruskal-Wallis test, followed by a *post hoc* multiple comparisons test (Wilcoxon rank-sum test with Bonferroni correction). Developmental stages labeled with different letters are significantly different (P < 0.05). (**c**) Modeling of bacteriocyte volume dynamics during aphid development. The volume of the bacteriocyte tissue per aphid was determined as the product of the bacteriocyte number by the bacteriocyte mean volume. A logistic model was applied for the first part of the dynamics, followed by an exponentially decreasing model. Volume increasing, *r*, and decreasing rates, *l*, are shown on the figure, with their 95% confidence interval. Shaded grey area indicates the laying period.

**Figure 7:**
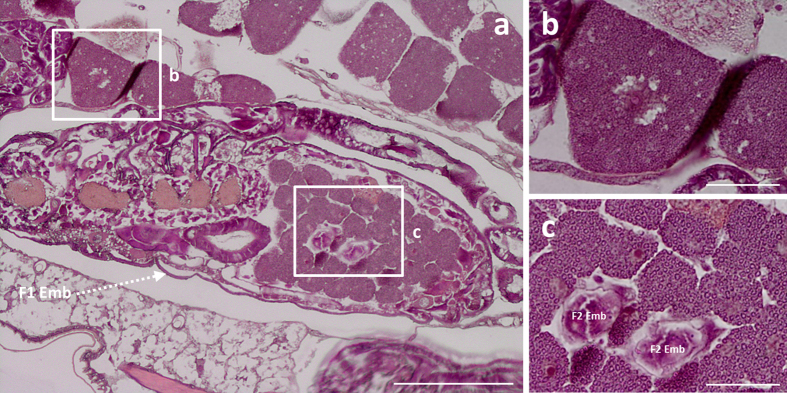
Histological comparison of bacteriocyte sizes between maternal and embryonic bacteriocytes in pea aphid nymphs. The hematoxylin and eosin staining performed on parthenogenetic N4 nymph sections (**a**) led to the clear identification of maternal (**b**) and late embryo bacteriocytes (**c**). Abbreviations: F1 Emb, embryos of the F1 generation; F2 Emb: embryos of the F2 generation (as described for the telescoping of generations in parthenogenetic aphids). Scale bars = 200 μm (**a**) or 50 μm (**b**,**c**).

In line with the endosymbiont dynamics in the maternal compartment, we observed a significant variation in bacteriocyte number during aphid development (Fig. 4a; Kruskal-Wallis test, P < 0.001). This analysis revealed a gradual increase during nymphal development, with the median number of bacteriocytes per aphid increasing from 34, at the LE stage, to reach 84 at the A9 adult stage. Such dynamics were confirmed by the modeling approach that gave rough estimations of the exponential growth and decreasing rates for the bacteriocytes (*r* = 0.32 ± 0.5 day^−1^ and *l* = 0.069 ± 0.05 day^−1^, Fig. 4b). Notably, this phase was characterized by the clustering of the bacteriocytes into two regular structures in the aphid abdomen (Fig. 5a). Once the nymphal development is complete, corresponding to the beginning of the laying period, the bacteriocyte structures start degenerating. We found a considerable decrease in the bacteriocyte number during aphid aging (2.3-fold from A9 to A23) associated with the dissociation of the structure (Fig. 5b). Additionally, we found that the bacteriocyte size changes significantly over the aphid life cycle (Fig. 6; Kruskal-Wallis test, P < 0.001) but with a different dynamics when compared to the variation of bacteriocyte number. This analysis, performed on more than 4,000 bacteriocytes, shows a long period of cell volume increase, from the LE embryo stage (2.2 × 10^4^ μm^3^) to the A13 time point (1.1 × 10^6^ μm^3^), followed by a stationary phase until the A16 time point. In the adult senescence phase, the dynamics ends with a significant drop in bacteriocyte size (A23: 5.9 × 10^5^ μm^3^). At this stage, we observed a reduction in bacteriocyte granular texture indicating a decrease in endosymbiont density (Fig. 5c,d). We confirmed these data regarding the variation of bacteriocyte size by performing histological analysis. As an example, a 2.9-fold diameter ratio was observed between N4 and LE bacteriocytes, localized in maternal and embryonic compartments, respectively (Fig. 7).

## Discussion

Here, we have developed a novel approach based on flow cytometry to quantify the dynamics of absolute cell numbers of *B. aphidicola*, the obligate endosymbiont of *A. pisum*, during the entire insect host life cycle. The experimental protocol is based on: (i) isolation of bacteria from other aphid tissues by filtration, (ii) cell staining by the SYTO9 nucleic acid dye and (iii) symbiont discrimination by FSC (related to cell size) and SSC (related to cell granularity) flow cytometry parameters. This resulted in a clear identification and quantification of *B. aphidicola* cells in whole aphids and in the embryonic chains they contain. The process was extremely efficient with only one major cell population identified, which represented more than 96% of the total events detected. Our results demonstrate the usefulness of flow cytometry for the quantitative analysis of endosymbionts extracted from fresh whole insects and their tissues. Importantly, the method permitted an efficient analysis of samples containing a wide range of endosymbiont numbers: from 1 × 10^4^ bacteria in the embryonic chains of the first nymphal stage to 1.2 × 10^7^ bacteria from the whole adult aphid body. Even with samples containing a low symbiont number, we did not notice any interference from electronic background noise and debris, two important confounding elements frequently encountered when flow cytometry is used to detect cells in low concentration samples. We attribute this to the optimized extraction procedure, which limits the presence of aphid cellular fragments, and the flow cytometry settings developed through this study, which avoid misleading signals, with less than 1.5% of the total events being SYTO9-negative. This protocol enabled us to measure samples containing only ten aphids, thus opening the way to establishing, in the future, large-scale analyses of aphid and other insect populations collected in the field, or complex laboratory experiments generating large numbers of insects following several parallel treatments.

Furthermore, the ease of this new approach, the speed of data processing and the relatively low reagent cost provide numerous advantages compared to alternative methods employed to evaluate the symbiotic load in specific aphid developmental stages. In fact, our quantification of endosymbionts is globally consistent with previously reported PCR^16^,^17^,^19^,^21^,^22^ or histology data^12^,^26^. PCR applications, estimating the number of *B. aphidicola* gene copies, are limited in that they do not allow for absolute endosymbiont quantification but provide only relative data through the comparison of various developmental or physiological conditions. Furthermore, the occurrence of multiple genome copy numbers in *B. aphidicola*, varying inside the symbiont population within the same aphid^39^ and also during insect development^40^, considerably limits the use of quantitative PCR in this symbiosis model. Flow cytometry overcomes these limitations by identifying endosymbionts according to cellular parameters (cell size and granularity) and allowing for direct cell number counting. While a stage-by-stage comparison between the present flow cytometry data and previous PCR results is not possible as none of those studies has analyzed the *B. aphidicola* dynamics throughout pea aphid development, our results show, overall, the same temporal dynamics as previous data using quantitative PCR techniques^16^,^17^. The *B. aphidicola* population increases during nymphal development, reaching a maximum in early adulthood, before progressively decreasing during aphid aging. However, the range of changes is different. When comparing day 5 and day 10 of the aphid life cycle, previous studies have reported a 5 to 8 fold-change while the present flow cytometry analysis shows a 24 fold-change in *B. aphidicola* cell number between these two time points. This discrepancy may be explained not only by the biases inherent to the PCR method but also by the differences in aphid treatments and genotypes. In fact, previous experiments did not analyze the dynamics of the primary symbiont population in standard physiological conditions. Sakurai *et al.*^17^ have investigated the impact of the presence of secondary symbionts *Rickettsia* spp. on the *B. aphidicola* population dynamics by comparing a *Rickettsia*-infected aphid strain OY and a *Rickettsia*-eliminated strain OY^amp^, following treatment with ampicillin. Similarly, Koga *et al.*^16^ have compared *B. aphidicola* cell dynamics between a *Serratia*-infected aphid strain, AIST^IS^, generated by haemolymph injections containing both *Serratia spp.* and *B. aphidicola* symbionts, and a *Serratia*-free aphid strain, AIST^AIST^, generated by haemolymph injections containing only primary symbionts. In both studies, the effects of aphid manipulation on the physiology of the primary symbiont cannot be excluded.

Our absolute cell numbers are generally in good agreement with previous studies. Mira and Moran^26^ have estimated, by a histological approach, that late embryonic stages and first nymphal instars contain 36,700 and 119,490 *B. aphidicola* cells, respectively. Our analysis revealed endosymbiont numbers of 25,900 and 61,060 bacteria for the same aphid developmental stages. Data from additional insect developmental stages are not available from Mira and Moran^26^. Humphreys and Douglas^41^ have reported, from a quantitative DNA hybridization experiment, that young adult aphids, reared at 20 °C, contain approximately 4 × 10^7^ *B. aphidicola* cells. We counted here 1.2 × 10^7^ *B. aphidicola* cells in young adult aphids maintained at 21°C.

By using flow cytometry, we also quantified the endosymbiont load in embryonic chains during the entire maternal life cycle. The absolute number of *B. aphidicola* cells appeared to increase with maternal development. In fact, as the parthenogenetic viviparous females grow, the embryonic chains gradually develop and they consist of more and more mature embryos^14^,^42^,^43^, which are, in turn, expected to contain an increasing number of symbiotic bacteria (i.e. endosymbionts, initially transferred in early embryos after blastoderm cellularization, are suspected of undergoing numerous division cycles during embryo maturation). Notably, this first phase of increase does not reach a maximum at the onset of the laying period but just afterwords, at day 13, when the embryonic chains encompass the mainly mature embryos. Then, the massive production of nymphs that occurs during adult life leads to a significant decrease in endosymbiont number, as we observed in embryonic chains during aphid aging. Interestingly, the few symbionts that we have counted in the embryonic chains of senescent aphids were due to the retention, by the mother’s body, of mature embryos that were never birthed.

**Figure 8:**
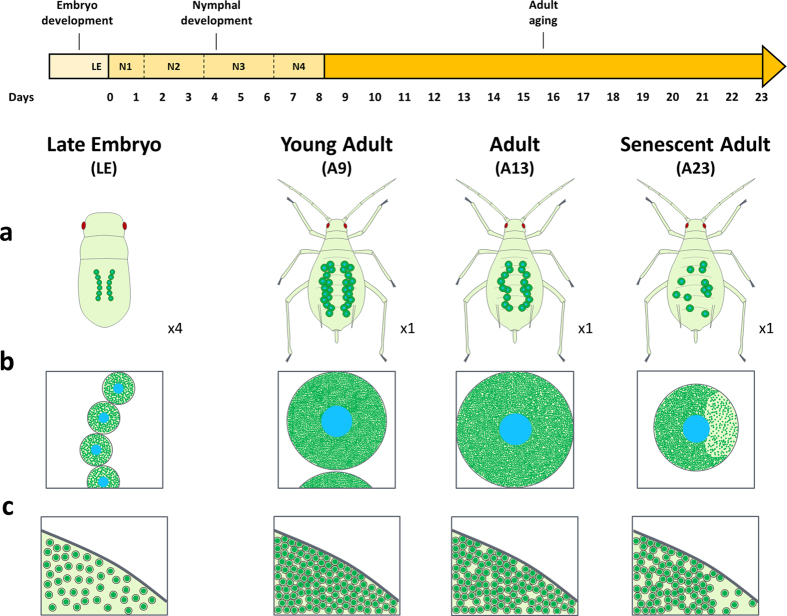
Schematic model of the dynamics of *B. aphidicola* and bacteriocyte cells during parthenogenetic development of the pea aphid. Evolution of bacteriocyte number and organization (**a**), bacteriocyte size (**b**), or symbiont density (**c**), in the pea aphid body, from the late embryo to the senescent adult. In each panel the scale is constant, except for panel (**a**) where the embryo is 4x compared to adult aphids.

Analysis of the symbiotic cells throughout aphid development, from the embryo to the senescent adult, reveals a coordination of the dynamics between the maternal bacteriocyte cells and the primary endosymbionts they contain (Fig. 8). A concomitant increase in both the *B. aphidicola* and the bacteriocyte numbers was observed during nymphal development, peaking at the onset of the adult stage. During this period, the bacteriocytes increase not only in number but also in size, clustering in two regular structures inside the aphid abdomen. Since the growth rate of the endosymbiont population (*r* = 0.85 day^−1^, Fig. 3e) is higher than the increasing rate of the volume of the bacteriocyte tissue in which they are localized (*r* = 0.76 day^−1^, Fig. 6c), this phase is also characterized by a considerable increase in *B. aphidicola* cell density within bacteriocytes. The multiplication of symbiotic cells during nymphal development coincides with a period of the aphid life cycle in which high metabolic activities are required, both for insect development (growth and molting processes) and the rapid development of large numbers of embryos in the embryonic chains. In the light of *B. aphidicola* functional specialization in aphid nutritional complementation^7^,^10^,^44^,^45^,^46^, the massive increase of primary endosymbionts observed during aphid nymphal development can be considered as an adaptive mechanism for supplying the amino acids and vitamins necessary to support this aphid embryonic and nymphal development^47^,^48^,^49^.

Once adulthood is reached and the laying period has started, the dynamics of symbiotic cells in aphids reverses (Figs. 3 and 4). Bacteriocyte structure degenerates and the number of bacteriocytes and primary endosymbionts progressively decreases as the aphid ages (Fig. 8). We interpret this as a correlation of a decreasing metabolic demand and of the disproportionate cost of maintaining a large endosymbiont population. Thus, the degeneration of the symbiotic structures may enable a balance to be reached between host physiological cost and endosymbiont benefits. It is noteworthy that, even in adult and senescent life cycle phases, we did not observe a complete elimination of the bacteriocytes and the endosymbionts they contain, but only a decline until a certain level, presumably that which is necessary for aphid survival. This age-dependent degeneration of symbiotic structures is even more pronounced in other insects. In carpenter ants only very few, if any, somatic bacteriocytes have been detected in old workers ants^18^,^50^. More dramatically, a total and rapid atrophy of somatic bacteriomes, associated with a complete elimination of the endosymbiotic population, has been observed in *Sitophilus* weevils rapidly after the adult emergence and cuticle synthesis^23^. By contrast to weevil symbiosis, where insects recycle endosymbionts when any benefits have expired, the maintenance of symbionts (and bacteriocytes) in old aphids reflects the high level of *Buchnera* integration in the physiology of their hosts. The long co-evolutionary history of aphids with *B. aphidicola* renders these bacteria profitable throughout the insect cycle, with their numbers being fine-tuned as a function of physiological demand.

The significant endosymbiont decrease in line with aphid aging is likely the result of two factors that can directly impact the symbiotic load. First, given that the histological analysis shows bacteriocyte structure degradation, we propose autophagy as a possible mechanism. Indeed, autophagy was recently demonstrated as regulating the proliferation of the parasitic reproductive endosymbionts *Wolbachia* spp., in *Drosophila* and filarial nematodes^51^, or the *Sodalis pierantonius* endosymbiont load, in weevils^23^. Secondly, in parallel with the reduction in the bacteriocyte size, we have also observed a decrease in *B. aphidicola* density in these cells. This phenomenon takes place after the final ecdysis, the developmental phase in which the lysosomal system in aphid bacteriocytes has been shown to trigger symbiont degradation^52^. Further cellular and molecular studies are needed to examine the precise involvement of cell death processes in the regulation of symbiont number in the aphid model.

In conclusion, this study sheds new light onto how symbiotic cells numbers, and bacteriocyte sizes and localization vary in response to host developmental needs within a nutritional insect symbiosis. Our results reveal a coordinated dynamics between the host cells, the bacteriocytes, and the primary endosymbionts they contain, providing a step-by-step picture from parthenogenetic embryos to the senescent adults. They highlight the need for identification of the cellular and molecular processes regulating these dynamics during the insect’s physiological development, as well as under stress conditions. The flow cytometry analysis developed in this study provides a promising approach for subsequent work on other symbiotic models, including for the elucidation of how multiple bacterial species are interacting with each other and with their host in the super-symbiotic systems, where a consortium of primary and secondary bacteria are living within the same eukaryotic organism.

## Methods

### Aphid rearing

A long-established parthenogenetic clone (LL01) of *A. pisum* Harris, containing only the primary endosymbiont *B. aphidicol*a, was maintained on young broad bean plants (*Vicia faba* L. cv. Aguadulce) at 21°C, with a photoperiod of 16 h light – 8 h dark. The absence of any of the secondary symbionts regularly occurring in pea aphids (*Hamiltonella defensa*, PAXS, *Regiella insecticola, Rickettsia* sp., *Rickettsiella* sp., *Serratia symbiotica*, *Spiroplasma* sp.) was checked by using the PCR-based diagnostic described by Peccoud *et al.*^53^. To obtain a source of synchronized aphids, winged adults were left on seedlings, allowing them to produce nymphs, and were removed after 24 h. Synchronized N1 nymphal instars were left to develop over 30 days. The survival curve of LL01 *A. pisum* clone in our temperature and photoperiod conditions shows that, after time point A23, less than 47% of the aphid population survived (see Supplementary Fig. S1 online).

### Sampling of synchronized aphids

For all experiments, aphids were collected at different developmental stages: late embryos LE (>0.8 mm), corresponding to embryonic stages 19–20 as described by Miura *et al.*^14^; nymphs N1 (first instars; 1 day old), N2 (second instars; 2 days old), N3 (third instars; 5 days old), and N4 (fourth instars; 7 days old); and adults at four distinct time points: A9 (9 days old), A13 (13 days old), A16 (16 days old) during reproductive period and A23 (23 days old) during the senescence period. All collected nymphs and adults were randomly selected from the synchronized source population. Late embryos or embryonic chains were collected by dissection from 13-day-old adults.

### Tissue dissection and extraction of endosymbionts by filtration

Symbiotic bacteria were purified from aphids using a protocol adapted from procedures previously described^48^,^54^,^55^. Aphid bacteriocytes or embryonic chains were carefully dissected in ice-cold isosmotic buffer A (0.025 M KCl, 0.01 M MgCl_2_, 0.25 M Sucrose, and 0.035 M Tris-HCl, pH 7.5) under 25X-40X magnification with a MDG-17 stereomicroscope (Leica, Wild Heerbrugg AG, Switzerland). For the complete experiment, ten whole aphids or ten embryonic chains, for each developmental stage, were gently crushed with a Potter apparatus in 3 ml of ice-cold buffer A. The homogenate was successively filtered through nylon net filters of 60, 30 and 10 μm pore sizes (Merck Millipore, Tullagreen, Ireland) and centrifuged (4,000 × g, 5 min, 4 °C). The resulting pellet, containing endosymbiotic bacteria, was re-suspended in 200 μl (LE to N2 stages) or 400 μl (N3 to A23 stages) of NaCl 0.85% supplemented with 20% glycerol as a cryoprotective agent, and stored at −80 °C until flow cytometry analysis. The cytometry profiles obtained on frozen cells were compared to those of fresh cells to preclude any cryoconservation effects. To remove abiotic particles of size and density similar to bacteria, buffer A was filtered at 0.22 μm prior to use. For each developmental stage, three independent biological replicates were processed.

### Fluorescence staining methods

The same staining procedure was applied for samples analyzed with flow cytometry or epifluorescence microscopy. Immediately prior to use, symbiotic bacteria samples, collected as described above, were thawed for 3 min at 42 °C, vortexed and diluted 1:20 with a NaCl 0.85% solution to reach the optimal cells concentration for flow cytometry analysis (< 2,000 events/sec). Nucleic acid probe SYTO9 from the LIVE/DEAD *Bac*Light bacterial viability kit (Molecular Probes Inc., Eugene, OR, USA) was used following the manufacturer’s instructions. Briefly, 0.5 μl of SYTO9 (3.34 mM) was added to 300 μl of the diluted bacterial cells suspension. The mixture was vortexed and incubated for 15 min at room temperature in the dark.

### Flow cytometry and microscopy analyses of endosymbionts

The stained samples were analyzed using a FACSCanto II flow cytometer (BD Biosciences, Erembodegem, Belgium) equipped with a blue laser (488 nm, air-cooled, 20 mW solid state). Forward-angle light scatter (FSC), indicating relative differences in cell size, and side-angle light scatter (SSC), indicating relative differences in cell granularity, were collected. Regardless of the stain used, cell green fluorescence was acquired with a photomultiplier tube detector and a 530 nm band-pass filter (515–545 nm). For each parameter (FSC, SSC and fluorescence), signals were converted to proportional voltage pulses defined by three waveform features: maximum intensity, H (height); time duration, W (width); and the integral of the pulse, A (area). Flow cytometry measurements were run at low flow rate (12 μl/min) and the core stream was allowed to stabilize for 30 sec prior to the 2 min acquisition. Cellular data were collected and processed using FACSDiva software (version 6.1.2, BD Biosciences). The analyses were performed using logarithmic gains and specific detector settings, adjusted on non-stained samples to eliminate the endosymbiont autofluorescence. To eliminate debris, from the analysis, the cells were first gated on SYTO9 fluorescence signals. Then, the endosymbiont population was identified by screening samples on FSC-W vs. FSC-H and SSC-W vs. SSC-H dot plots.

SYTO9-stained symbionts were mounted onto slides and analyzed with an inverted epifluorescent IX81 Olympus microscope (Olympus Corporation, Tokyo, Japan) equipped with a specific emission filter, HQ535/50, for green signals. Microscopy images were captured at 160X magnification using an F-view camera linked to the CellF software (Soft Imaging 197 System).

### Counting and size determination of aphid bacteriocytes

For bacteriocyte counting, aphids were analyzed one by one. Bacteriocytes were surgically isolated from the abdomen of each individual aphid in ice-cold buffer A and counted at 25X-40X magnification with a MDG-17 stereomicroscope. To avoid counting several times the same cell, the bacteriocytes were eliminated one by one, after counting, with a Pasteur pipette attached to a vacuum pump. For each developmental stage, a total number of 20 aphids was analyzed.

To determine their size, bacteriocytes were collected with a micropipette and mounted on glass slides, using spacers cut from coverslips to avoid crushing cells. Bacteriocyte images were acquired with an IX81 Olympus microscope at 16X magnification. As previously described^56^, bacteriocytes appeared as irregular spherical cells. To obtain an estimate for cell volume, each bacteriocyte was thus treated as a sphere. The bacteriocyte area (A) was determined using the CellF software, and the bacteriocyte volume value (V) was calculated applying the standard formula: 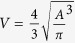. The choice of the variable “area”, instead of other variables like the cell diameter, aimed at obtaining a robust approximation of the bacteriocyte volumes even for the most irregular cells. For each developmental stage, 10–15 aphids were analyzed.

### Histology

To confirm the dynamics of bacteriocyte (size, localization and morphology), aphid tissue structures were analyzed at each developmental stage following the eosin/hemalum coloration previously described^57^. Antennae and legs were removed from aphids prior to fixation in a solution of 4% paraformaldehyde in phosphate buffered saline (PBS). After one week at 4 °C, the fixative solution was eliminated with several washes in PBS. To facilitate manipulation, samples were stained in 1% eosin for 1 min and embedded in 1.3% agar. Afterwards, samples were dehydrated through a series of ascending ethanol solutions and then moved to butanol-1 at 4 °C, for one day. Next, aphids were embedded in melted Paraplast (Mc Cormick Scientific LLC, St Louis, USA) and wax blocks stored in a dust-free environment. Tissue sections of 3 μm were cut using a LKB Historange microtome (LKB Instruments, Bromma, Sweden), placed on polylysine coated slides, dried overnight at 37 °C, and conserved at 4 °C until staining. Paraffin sections were de-waxed in methylcyclohexane, rinsed in absolute ethanol, and rehydrated through an ethanol series to PBS. Staining was performed using RAL products (REACTIFS RAL Martillac, France), according to the manufacturer’s instructions and sections were mounted in Mountex mounting medium (Histolab, Gothenburg, Sweden). Observations were performed under transmitted light, using an IX81 Olympus microscope at 10X-50X magnification.

### Statistics

All statistical analyses were carried out using the R software R v3.1.1, with values of P < 0.05 considered significant. As data did not meet the requirement of normality and homogeneity of variances (Shapiro-Wilk and the Bartlett tests), a log-transformation was performed. Log-transformed data of symbiont numbers were then analyzed using the one-way analysis of variance (ANOVA), followed by *post hoc* multiple comparisons using the Tukey’s HSD test. Log-transformed data of bacteriocytes (numbers and volumes) were not consistent with normality and homoscedasticity, so the nonparametric Kruskal-Wallis test was used to compare non-transformed data obtained for the different developmental stages, followed by *post hoc* multiple comparisons using the Wilcoxon rank-sum test with a Bonferroni correction.

Logistic 
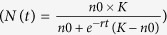
 and exponential decreasing 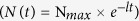 models were used to describe the dynamics of the *B. aphidicola* numbers and bacteriocyte numbers and volumes. The n0 parameters of the logistic models were fixed at the mean of the first observed values (LE stage) to reduce the variability of the growth rate estimations, *r*. Rupture time points were visually determined and the predicted values of the logistic models at these points were used to fix N_max_ in the decreasing exponential model, ensuring continuity and improving the estimation of the decreasing rate, *l*. Non-linear least-square fittings (Gauss-Newton algorithm) were realized using the nlstools R library; *r* and *l* values are given with their 95% confidence interval assuming normality of the parameter estimations.

## Additional Information

**How to cite this article**: Simonet, P. *et al.* Direct flow cytometry measurements reveal a fine-tuning of symbiotic cell dynamics according to the host developmental needs in aphid symbiosis. *Sci. Rep.* **6**, 19967; doi: 10.1038/srep19967 (2016).

## Supplementary Material

Supplementary Information
