# Supplementary material for: Direct flow cytometry measurements reveal a fine-tuning of symbiotic cell dynamics according to the host developmental needs in aphid symbiosis
Source: Sci Rep. 2016 Jan 29;6:19967. doi: 10.1038/srep19967 (PMC4731799; doi:10.1038/srep19967)

Direct flow cytometry measurements reveal a fine-tuning of symbiotic cell dynamics according to the host developmental needs in aphid symbiosis

Pierre Simonet[1]*, Gabrielle Duport[1], Karen Gaget[1], Michèle Weiss-Gayet[2], Stefano Colella[1], Gérard Febvay[1], Hubert Charles[1], José Viñuelas[1], Abdelaziz Heddi[1] and Federica Calevro[1]*

[1] UMR203 BF2I, Biologie Fonctionnelle Insectes et Interactions, INRA, INSA de Lyon, Université de Lyon, F-69621 Villeurbanne, France.
[2] UMR5534, Centre de Génétique et de Physiologie Moléculaire et Cellulaire, Université Lyon 1, CNRS, F-69622 Villeurbanne, France.

*Corresponding authors: pierre.simonet@insa-lyon.fr and federica.calevro@insa-lyon.fr

Tel.: +33 472 43 79 88

Fax: +33 472 43 85 34

Supplementary Information

Supplementary Figure S1. Survival of aphids in standard rearing conditions. Survival rate of aphids reared on *Vicia faba* L. plants, at 21°C, with a photoperiod of 16 h light – 8 h dark. Throughout the experimental period, aphid survival was monitored daily. n = 30 aphids (at day 0).

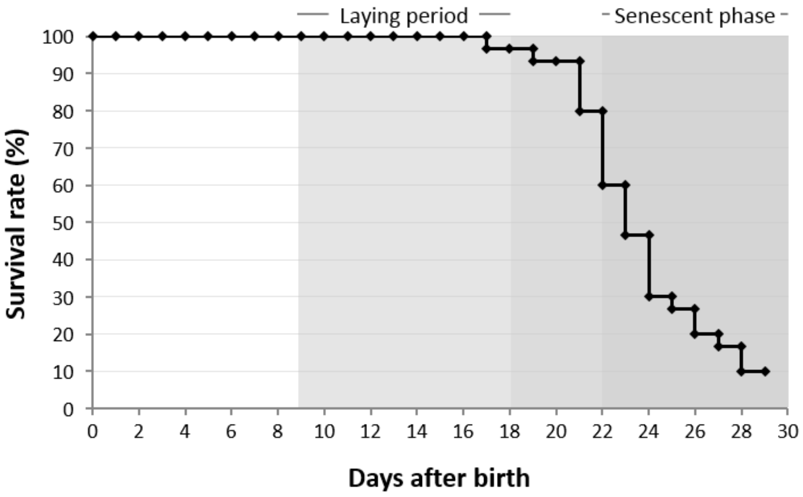

Supplement: Supplementary Information [file srep19967-s1.pdf]
